# A novel cause for primordial dwarfism revealed: defective tRNA modification

**DOI:** 10.1186/s13059-015-0786-y

**Published:** 2015-10-01

**Authors:** Liudmila Filonava, Adrian Gabriel Torres, Lluis Ribas de Pouplana

**Affiliations:** Institute for Research in Biomedicine (IRB Barcelona), Carrer de Baldiri Reixac 10, Barcelona, 08028 Catalonia Spain; Catalan Institution for Research and Advanced Studies (ICREA), Passeig de Lluis Companys 23, Barcelona, 08010 Catalonia Spain

## Abstract

A mutation in the *WDR4* gene, coding for a tRNA-modifying enzyme, leads to reduced levels of guanosine methylation in tRNA in patients with primordial dwarfism.

See related Research article: http://www.genomebiology.com/2015/16/1/210.

## Background

Transfer RNAs (tRNAs) have a key role in protein synthesis. They act as adaptor molecules capable of reading mRNA codons so as to deliver amino acids to the growing polypeptide in the translating ribosome. To function properly, tRNAs need to be chemically modified post-transcriptionally [1]. Some of the modifications help tRNAs to maintain a distinctive tertiary structure, others are critical for correct aminoacylation, and yet others participate in codon reading, thus influencing efficiency and fidelity of translation. Dysregulation of the tRNA modification pattern, or of the levels of the enzymes that catalyze these modifications, drastically alters tRNA functionality and can result in complex phenotypes, probably due to defects in protein translation [2].

Evidence is accumulating that tRNA modifications have significant roles in human diseases [3]. However, in many cases, although the link between tRNA modifications and pathology has been confirmed, the exact molecular mechanism behind the observed phenotypes remains unclear. In a new study published in *Genome Biology*, Shaheen and coworkers [4] describe a mutation in the human *WDR4* gene as a cause of primordial dwarfism (PD). This gene encodes a tRNA methyltransferase and has previously been indirectly associated with neurological disorders such as Down syndrome [5]. The work by Shaheen and colleagues [4] provides the first direct evidence for the role of WDR4 in a human disease, and it addresses the molecular mechanisms behind PD from a tRNA modification perspective.

## Towards understanding tRNA modification-driven pathogenesis

PD is an incurable spectrum of clinical conditions caused by various mechanisms, such as impaired DNA damage repair or impaired mitosis due to centrosomal abnormalities. It is characterized by growth deficiency, brain malformation and facial dysmorphism. Although some of the genetic causes for PD have been studied in depth, the molecular mechanisms behind many forms remain unknown.

*WDR4* maps to human chromosome 21q22.3, a region that has previously been associated with several genetic disorders, such as autosomal-recessive deafness, manic-depressive psychosis and Down syndrome [5]. Moreover, this chromosomal region has also been investigated in mice, where it was found that its copy number directly affects cognition and hippocampal plasticity [6]. Furthermore, the human *WDR4* gene has been found to have two splice forms, one of which is mainly expressed in fetal tissues, suggesting a potential role for this transcript in neural tissue development [5].

*WDR4* encodes one of the subunits of the heterodimeric enzyme that is responsible for methylating carbon 7 of guanosines at position 46 (m^7^G_46_) of nine human tRNAs. Its binding partner is METTL1, and the yeast homologs of WDR4 and METTL1 are Trm82 and Trm8, respectively. Lack of Trm82 or Trm8 leads to decreased levels of m^7^G_46_ in tRNA^Phe^ and results in a temperature-sensitive growth phenotype when cells are grown in synthetic media containing glycerol. Importantly, in yeast, human WDR4 and METTL1 can compensate for the lack of Trm82 and Trm8, respectively, suggesting a conserved role for these proteins and for the m^7^G_46_ modification in eukaryotes [7].

Shaheen and coworkers [4] worked with two consanguineous families with members affected by a distinct form of PD. Using autozygosity mapping and exome sequencing they identified a homozygous mutation in the *WDR4* gene in the affected family members. Moreover, lymphoblastoid cell lines derived from these patients showed decreased levels of m^7^G_46_. To further investigate the molecular effect of this mutation, Shaheen and coworkers [4] created a model yeast strain bearing the equivalent of the mutation found in human patients with PD, in the *trm82* gene (*Trm82-K223L*). As expected, the level of m^7^G_46_ modification was decreased in the *Trm82-K223L* mutant strain. Furthermore, the mutant yeast strain had a temperature-sensitive growth phenotype similar to that observed when the *trm8* gene was deleted [8]. Given that Trm8 and Trm82 can catalyze guanosine methylation only if they form a complex, the data suggested that the mutation disrupts an important interaction between Trm8 and Trm82. Trm82 is the non-catalytic subunit of the methyltransferase and serves to maintain the levels of Trm8 by stabilizing it in an active conformation [9]. Therefore, one possibility is that the mutation in human WDR4 affects its binding to METTL1, resulting in a reduction of m^7^G_46_ due to impaired formation of the WDR4–METTL1 heterodimer. However, a direct link between these observations and the severe phenotypic consequences observed in PD patients still needs to be made.

The finding that a point mutation in the non-catalytic subunit of an enzyme leads to complex phenotypes is reminiscent of the enzyme hetADAT (heterodimeric adenosine deaminase acting on tRNA), which deaminates A34 into inosine 34 (I34) in all tRNAs with the anticodon starting with A (tRNAANN). This enzyme is composed of two subunits, ADAT2 (the catalytic subunit) and ADAT3 (a non-catalytic partner) [8]. Both *ADAT2* and *ADAT3* are essential genes, although human cell lines can tolerate to some extent the reduction in I34 levels induced by ADAT2 knockdown [9]. The same group that now reports the *WDR4* PD mutation [4] previously reported a homozygous missense mutation in *ADAT3* to be associated with intellectual disability and strabismus [10]. Molecular modeling of the part of ADAT3 containing the mutated residue showed an altered structure of the loop where the mutated residue is located [10]. Potentially, this altered conformation could disturb the ADAT2–ADAT3 heterodimer and/or tRNA binding. Mechanistically, it is possible that the mutations in *WDR4* and in *ADAT3* both result in similar consequences for heterodimer formation and catalysis, but the effect of the lack of these modifications on the functionality of tRNA remains to be addressed in depth.

## Open questions and concluding remarks

The above-mentioned studies contribute to the exciting, but still poorly explored, issue of the role of tRNA modifications in human diseases. The findings of Shaheen and coworkers [4] raise several questions. How does the altered pattern of tRNA modifications influence the physiological functionality of tRNAs (structure, aminoacylation, tRNA recognition by the ribosome, codon decoding, and so on)? Does a lack of tRNA modifications lead to overall protein translation defects or does it affect only some particular genetic programs? Could non-canonical functions of tRNAs be altered in these pathological scenarios? Are there any more tRNA modifications associated with other human diseases? These and other questions are attractive research paths that deserve closer attention.
